# South African undergraduate students’ perceptions of clinical supervision in speech-language therapy

**DOI:** 10.4102/sajcd.v73i1.1146

**Published:** 2026-05-30

**Authors:** Avuzwa Makiva, Anathi Phandle, Nosipho Maphanga, Bongiswa Vukethwele, Dharshini Naidoo, Samantha Bassingthwaighte

**Affiliations:** 1 Department of Natural and Rehabilitative Sciences, Faculty of Health Sciences, University of Fort Hare, East London, South Africa

**Keywords:** speech-language therapy, clinical supervision, student perspectives, South Africa, qualitative research

## Abstract

**Background:**

Clinical training is a cornerstone of speech-language therapy education, bridging theory with practice. Effective clinical supervision is critical for developing students’ competence, confidence and professional identity. However, there is limited research on students’ perceptions of clinical supervision in resource-constrained settings, such as South Africa.

**Objectives:**

This study explored student speech-language therapists’ perspectives on clinical supervision during placements, identifying both facilitators and barriers to practical learning, with the aim of informing improvements to training quality and professional preparedness.

**Method:**

A qualitative exploratory research design was used, where semi-structured interviews were conducted with third- and fourth-year speech-language therapy students from a South African university. Participants (*n* = 14) were recruited through purposive sampling and data were analysed thematically.

**Results:**

Students emphasised that consistent supervision, constructive feedback and structured tutorials fostered improved skill development and confidence. Barriers included infrequent supervision, limited resources and unclear expectations. The emotional support provided by supervisors also significantly influenced students’ experiences.

**Conclusion:**

Strengthening clinical supervision through structured feedback, resource access and emotionally supportive student–supervisor relationships can improve speech-language therapy students’ learning and professional development. These insights have implications for improving supervision frameworks in South Africa’s under-resourced clinical training contexts.

**Contribution:**

This study provides context-specific insights into speech-language therapy students’ lived experiences of clinical supervision, reinforcing the need for equitable, supportive and standardised supervisory practices in South African higher education training settings.

## Introduction

Speech-language therapy is a healthcare field focused on assessing, diagnosing and managing communication disorders, including speech, language, fluency and swallowing impairments (Bailey, 2021). Speech-language therapists play a critical role in enhancing communication abilities, thereby improving the quality of life for their clients (Pollens, 2020). Beyond clinical practice, speech-language therapists contribute to research, education and policymaking, advocating for individuals with communication disorders and ensuring equitable access to services (Saxon et al., 2014). Given the broad scope of speech-language therapy practice, comprehensive clinical training and supervision are essential for students during their 4-year university degree programmes in South African institutions. Clinical supervision bridges the gap between theoretical knowledge and practical application, and comprises structured experiences that enable students to apply their knowledge in real-world settings (King et al., 2020; Rocha et al., 2020; Snowdon et al., 2020). This training typically follows a tiered approach, progressing from observation to direct patient care under supervision, within diverse speech-language therapy contexts through strategic clinical placements (Rocha et al., 2020). Clinical placements occur in various settings, including hospitals, schools, rehabilitation centres and community clinics, offering students exposure to diverse populations and clinical challenges (Hardy et al., 2021; Pope et al., 2023). These placements aim to develop professional behaviours, critical thinking and technical skills necessary for effective practice within the broad scope of the speech therapy field (Hardy et al., 2021; Sheepway et al., 2014; Snowdon et al., 2020). Clinical training also offers opportunities for students to gain insight into the multidisciplinary nature of healthcare, learning to collaborate with professionals from different fields to deliver holistic, patient-centred care (Wilson et al., 2016). Structured clinical supervision is an essential component of training to ensure that students are adequately supported and are able to benefit fully from these diverse clinical experiences. It involves guidance, access to resources and mentorship provided to students during their clinical placements. This includes direct observation, constructive feedback and skill demonstration by experienced clinicians (O’Brien et al., 2019; Quigley et al., 2020; Rocha et al., 2020). Supervision fosters a safe environment for professional growth, enabling students to navigate challenges with structured support (O’Brien et al., 2019). Adequate clinical supervision is crucial for building students’ confidence, competence and preparedness for independent practice (Quigley et al., 2020). Supervisors are expected to provide structured feedback, model professional behaviour and promote reflective practice (Quigley et al., 2020). They also facilitate interdisciplinary collaboration, preparing students to work effectively in team-based healthcare settings (Yang et al., 2017). Furthermore, supervisors guide students in developing essential clinical and interpersonal skills such as communication, problem-solving and cultural competence (Attrill et al., 2015; Quigley et al., 2020). *Clinical* supervisory roles in speech-language therapy are particularly complex in South African clinical education because of the country’s linguistic diversity and persistent healthcare inequities (Pillay & Kathard, 2018). Students frequently undertake clinical placements in multilingual and under-resourced contexts where supervisors must guide students in culturally and linguistically responsive practices (Khoza-Shangase & Mophosho, 2021). Within this context, cultural responsiveness cannot be understood solely as interpersonal sensitivity or awareness of diversity. Clinical education and supervision are embedded within broader sociohistorical conditions shaped by the enduring legacies of Apartheid, which continue to influence educational access, institutional resources, professional hierarchies and power relations (Khoza-Shangase & Mophosho, 2021; Pillay & Kathard, 2018;). Research emphasises that culturally responsive practice in South Africa requires engagement with systemic and institutional factors, rather than remaining solely at the level of individual adaptation (Khoza-Shangase & Mophosho, 2021; Pillay & Kathard, 2018). Recent empirical work further highlights the persistence of structural, cultural and linguistic training gaps within speech-language therapy education, with students reporting limited Afrocentric curriculum content, insufficient integration of South African languages, and perceived underpreparedness for working with linguistically diverse populations (Abrahams & Khoza-Shangase, 2025). These findings suggest that supervision should be understood within the broader institutional and systemic contexts shaping clinical education, rather than being viewed solely as an interaction between supervisor and student. Consistent with this, many speech-language therapy students encounter additional challenges during clinical training that may hinder their professional development (Mupawose et al., 2021; Nagdee et al., 2022; Weallans et al., 2022). Existing research highlights barriers experienced by students, including limited access to resources, inconsistent feedback and high workloads (Mupawose et al., 2021). Resource constraints, such as shortages of assessment tools, therapy materials and technological infrastructure, may restrict learning opportunities and compromise the quality of intervention provided during training (Nagdee et al., 2022). Furthermore, variability in supervision quality and inconsistency of feedback may impede students’ ability to reflect on and address areas of clinical improvement (Weallans et al., 2022). Understanding how students develop competence under such constraints requires a theoretically grounded perspective on learning. From a constructivist perspective, students develop clinical competence through active engagement with experiences, feedback and guided reflection rather than passive knowledge transmission (Biggs & Tang, 2014). Experiential learning theories, particularly Kolb’s (1984) experiential learning cycle, provide a useful lens for understanding how learners transform clinical experiences into professional knowledge through cycles of concrete experience, reflective observation, conceptualisation and active experimentation (Kolb, 1984). Within this framework, clinical supervision functions as a critical pedagogical mechanism through which these learning processes are scaffolded, as supervisors structure experiences, facilitate reflection and guide the integration of theory and practice (Terry et al., 2020). To better understand and address these challenges within a South African context, this study is guided by the Proctor Model (Proctor, 1986) of supervision, which conceptualises clinical supervision as comprising three interrelated functions: formative, normative and restorative (Inskipp & Proctor, 2009; Proctor, 1986). These functions correspond to students’ clinical, ethical and emotional support needs throughout their training. Formative supervision focuses on developing clinical competence, decision-making and reflective learning. Normative supervision emphasises professional accountability, ethical standards and policy adherence. Restorative supervision provides emotional support and promotes well-being (Inskipp & Proctor, 2009; Litherland et al., 2023). While Proctor’s model (Proctor, 1986) provides a widely adopted framework for conceptualising supervision, South African literature highlights persistent challenges within supervisory practices that reinforce its continued relevance (Hendricks et al., 2021). Hendricks et al. (2021) note that clinical supervision in South Africa is frequently characterised by inconsistency, resource constraints and supervisors with limited formal training. Within this evolving landscape, Proctor’s functional framework remains particularly valuable in contexts where supervision must simultaneously support student learning, uphold professional standards, and foster emotional well-being within resource-constrained training environments. In South Africa, clinical training occurs within a healthcare system marked by significant inequities in service access, workforce shortages and high patient caseloads (Müller et al., 2022; Wille & Maqbool, 2023). These challenges are further compounded in the Eastern Cape, one of the country’s most under-resourced provinces, characterised by vast rural areas, limited rehabilitation facilities, linguistic and cultural diversity, and socioeconomic disparities that affect both healthcare delivery and student learning opportunities (Wille & Maqbool, 2023). Within this complex environment, supervisors must balance service delivery demands with the responsibility of mentoring students, often with limited institutional and material support. Given these pressures, supervision must extend beyond instructional and evaluative roles to include supporting students as they navigate emotionally demanding and resource-limited clinical contexts. Within the Proctor Model (Proctor, 1986), this responsibility aligns most closely with the restorative function, which emphasises the supervisor’s role in promoting supervisees’ emotional well-being, resilience and professional stamina, while operating in concert with the formative and normative functions to ensure ongoing learning, ethical practice, and reflective growth. The Health Professions Council of South Africa (HPCSA) mandates that graduates must manage the full scope of practice and adapt their assessment and intervention methods for multilingual populations. This is particularly crucial during community service, where supervision may be limited, and caseloads are high. To meet these regulatory demands, strong theoretical preparation and robust, high-quality clinical supervision are essential. However, supervisors have reported that many students enter clinical placements lacking sufficient integration of theory and practice, which undermines their clinical confidence and competence (Mupawose et al., 2021). Furthermore, the limited availability of culturally and linguistically appropriate assessment materials, combined with minimal training in cultural competence, leaves students underprepared for working with multilingual clients (Abrahams & Khoza-Shangase, 2025). This gap between regulatory expectations and the realities of clinical training highlights the need to examine how clinical supervision can better support student readiness in South Africa. In response to these concerns, this study explores speech-language therapy students’ perspectives on clinical supervision during training in South Africa. Although existing literature highlights the critical role of clinical support in developing professional competencies, a few qualitative studies have captured the lived experiences of speech-language therapy students in the South African context. This study seeks to address this gap by amplifying student voices to guide improvements in supervision and support in clinical training programmes in the unique context of South Africa.

## Research methods and design

The objectives were:

### Objectives

- To explore speech-language therapy students’ perspectives on clinical supervision during their clinical training.
- To identify the barriers and facilitators experienced by students with regard to clinical supervision during their clinical training.

### Study design

This study employed a qualitative exploratory research design to examine speech-language therapy students’ perceptions of the clinical supervision experienced during their clinical placements. This allowed access to students’ lived, subjective experiences, ensuring rich, contextualised narratives that may not otherwise be captured through quantitative methods (Creswell & Poth, 2016).

### Setting

The study was conducted in the Department of Speech-Language Pathology at the University of Fort Hare. The students in this university are placed in various clinical training sites, including public hospitals, schools and community health centres, all of which reflect the linguistic and socioeconomic diversity of the province.

### Study population and sampling strategy

Participants included 14 undergraduate speech-language therapy students who were actively engaged in clinical training as part of their degree requirements. The sample comprised 14 participants: third-year (*n* = 6) and fourth-year (*n* = 8). Participants ranged in age from 21 years to 23 years. The group included eight female and six male students. All participants self-identified as black South Africans. Inclusion criteria required that students had completed at least one clinical rotation. Students who had not yet begun their placements, were on academic leave, or were outside the specified years of study, were excluded. Non-probability purposive sampling was used to ensure that participants had direct experience with the clinical training environment (Creswell & Creswell, 2017). Data collection and analysis occurred iteratively, consistent with qualitative approaches to data saturation, whereby themes are refined as interviews progress and recruitment continues until no new insights emerge (Guest et al., 2020). The adequacy of this sample size was informed by the depth of the emerging data and the relative homogeneity of the group, which reduces the variability that typically requires larger samples. This aligns with the observation of Guest et al. (2020), who found that in relatively homogeneous samples, thematic saturation is often reached within the first 12 interviews. However, this was used only as a guiding benchmark rather than a prescriptive number; saturation in this study was determined through ongoing comparison of codes, categories and thematic patterns, not solely by predetermined sample thresholds.

### Data collection

Following ethical clearance, gatekeeper permissions, and acquisition of informed consent, data collection commenced. Data were equally collected by two fourth-year SLT students who acted as peer interviewers. Peer interviewers were not included in the study as participants. Peer interviewing can facilitate openness and reduce hierarchical barriers; however, it also introduces risks such as familiarity bias, shared assumptions and variability in interviewer style (Byrne et al., 2015). To mitigate these concerns, both interviewers used a procedural interviewing checklist that standardised introductions, question delivery, probing and closing procedures, ensuring consistency across interviews and reducing the likelihood of over-rapport or uneven depth between interviewers. The semi-structured interview guide used in this study followed Creswell and Creswell’s (2017) guidance that qualitative interviews should consist of broad, open-ended questions that enable participants to construct the meaning of their experiences in their own terms. Likewise, Creswell and Creswell (2017) state that the interview protocol should include 5–10 open-ended content questions with accompanying probes, prepared in advance and used consistently across interviews. In keeping with this approach, the questions in this study were intentionally designed to be flexible rather than to mirror each specific category of Proctor’s (Proctor, 1986) formative, normative and restorative functions. Instead, the open-ended format allowed participants to discuss whichever aspects of clinical supervision were most meaningful to them, while still providing opportunities to address learning processes, expectations and emotional experiences. The full interview guide and a summary showing how each question offered space for discussion related to Proctor (Proctor, 1986) domains are included in Online Appendix 1. Interviews were conducted either face-to-face or telephonically, depending on participant preference and lasted between 45 min and 60 min. Although interview mode can influence interactional dynamics, evidence indicates that telephonic interviews can produce data comparable in rapport, depth, and richness to face-to-face interviews when supported by consistent questioning, clear articulation, and active listening strategies (Farooq & De Villiers, 2017). These recommendations informed the procedures used in this study; both modes followed the same interview guide and checklist. Participants were reminded that peer interviewers held no evaluative authority and that confidentiality was assured, minimising impression-management and supporting voluntary participation. Interviews were conducted in English, audio-recorded with consent and transcribed verbatim. An anonymisation protocol was implemented using coded identifiers (e.g. ‘Participant 3’) to ensure confidentiality. A pilot interview was conducted with one participant (approximately 10% of the sample) to refine the interview guide. No significant changes were required, and the data from this interview were included in the final analysis.

### Data analysis

Thematic analysis was conducted following the six-phase framework of Braun and Clarke (2006), using an inductive, data-driven approach. The researchers, who also conducted the peer interviews, independently familiarised themselves with the transcripts and generated initial codes. This independent coding reflects investigator triangulation, a strategy shown to reduce individual researcher bias and strengthen credibility by incorporating more than one analytic perspective (Nowell et al., 2017). After coding separately, the researchers met to compare interpretations, discuss discrepancies, and reach consensus on the coding framework. This collaborative process aligns with recommended practices for qualitative analysis teams, where shared interpretation and negotiated decision-making enhance analytic rigour and dependability (Richards & Hemphill, 2018). Manual coding was used to remain close to the data and preserve contextual sensitivity. A clear audit trail, including coding notes and records of analytic meetings, was maintained to support transparency and methodological coherence (Nowell et al., 2017). The thematic analysis process is shown in Table 1.

**TABLE 1:** Data analysis process.

Phase (Braun & Clarke, 2006)	Application to study
1. Familiarisation with the data	Both researchers independently read transcripts multiple times and captured early impressions.
2. Generating initial codes	Manual coding across all transcripts. Codes were compared and harmonised across analysts.
3. Searching for themes	Codes were grouped into preliminary themes and subthemes using collaborative consensus.
4. Reviewing themes	Themes were refined to ensure internal coherence and distinctiveness.
5. Defining and naming themes	Themes and subthemes were defined and named to reflect conceptual meaning.
6. Producing the report	Themes were written up with supporting extracts and mapped onto Proctor’s model (Proctor, 1986).

Note: Please see the full reference list of the article, Makiva, A., Phandle, A., Maphanga, N., Vukethwele, B., Naidoo, D., & Bassingthwaighte, S. (2026). South African undergraduate students’ perceptions of clinical supervision in speech-language therapy. *South African Journal of Communication Disorders*, *73*(1), a1146. https://doi.org/10.4102/sajcd.v73i1.1146, for more information.

### Trustworthiness

Trustworthiness was upheld through strategies addressing credibility, dependability, transferability and confirmability (Babbie, 2020). Credibility was strengthened through investigator triangulation, with two researchers independently analysing all transcripts before resolving differences through consensus discussions. Member checking was also implemented, where participants were given the opportunity to verify the accuracy of their transcripts and clarify their intended meanings (McKim, 2023). These approaches align with recommendations for enhancing the robustness and trustworthiness of thematic interpretations (Nowell et al., 2017). Dependability was supported through a detailed audit trail documenting coding decisions, theme development, and analytic discussions, consistent with best-practice guidelines for transparent qualitative analysis (Richards & Hemphill, 2017). Transferability was facilitated by providing rich descriptions of the clinical training context and participant characteristics. Confirmability was reinforced through ongoing discussion of assumptions during analytic meetings, ensuring that interpretations remained grounded in participants’ accounts rather than researcher bias (Nowell et al., 2017).

### Ethical considerations

Ethical clearance to conduct this study was obtained from the University of Fort Hare Health Research Ethics Committee (Ethics clearance number: REC-100118-054; Certificate reference number: 024=06=26 HRECLVN08). All participants provided written informed consent. Confidentiality was maintained through the anonymisation of transcripts and secure data storage.

## Results

Three interrelated themes emerged from the analysis: (1) facilitators of clinical supervision, (2) barriers to clinical supervision, and (3) student experiences of clinical supervision. These themes represent students’ (*n* = 14) lived experiences of supervision, resource availability, and emotional support within clinical placements. The themes and subthemes are described below with supporting quotations.

### Theme 1: Facilitators of clinical supervision

Students described four supervisory practices and environmental factors that enhanced their learning and contributed positively to their clinical development. These facilitators included constructive feedback, preparatory tutorials, access to clinical resources and supportive supervisory relationships. Together, these elements provided students with clarity, confidence and a stronger sense of direction during clinical placements.

#### Constructive feedback

Students (*n* = 8) consistently valued feedback that was specific, actionable, and delivered in a supportive manner. They highlighted that feedback helped them recognise mistakes, refine assessment and therapy procedures, and improve their clinical reasoning:

‘She’ll watch you … and if she sees that you are doing something wrong, that’s when she will provide support … give you feedback, give you recommendations of what you can do next, and provide you with resources you can use. For instance, in aphasia, she recommended workbooks we can use for aphasia patients.’ (Participant 11, female, student SLT)

#### Structured tutorials and preparation

Students (*n* = 6) emphasised the usefulness of tutorials provided before and during placements. Tutorials strengthened their ability to link theory with practice and helped them feel more prepared for diverse clinical contexts:

‘Before going to the clinical sites, we had tutorials … they taught us what to do and helped us reflect at the end of the day.’ (Participant 9, female, student SLT)

#### Access to clinical resources

Students (*n* = 8) described how access to updated assessment tools, clinical articles and standardised protocols enhanced their competence and confidence in assessment and intervention:

‘There were a lot of materials given … the CELF, TAPS, Articulation Assessment, TELD, the TACL, and these helped a lot in improving how I assessed a patient.’ (Participant 13, male, student SLT)

‘We were given the CELF articles to read and the protocols we had not been exposed to … they explained each sub-area.’ (Participant 1, male, student SLT)

#### Supportive supervisory presence

The relational qualities of supervisors, such as patience, approachability and consistent availability, were frequently cited as sources of reassurance and motivation by nine students:

‘My supervisor was amazing … She was patient and gave me information I never knew about. She made things easier for me.’ (Participant 10, female, student SLT)

Overall, these facilitators strengthened students’ readiness, confidence and clinical skill development.

### Theme 2: Barriers to clinical supervision

Students described three prevalent challenges that hindered their learning during clinical placements. These included inconsistent supervision, high supervisor workload and limited access to clinical materials. These barriers often led to feelings of uncertainty, underpreparedness and frustration.

#### Inconsistent or limited supervision

Eight student participants reported infrequent supervision, leading to uncertainty when managing clinical cases independently. Students felt that this restricted their opportunities to ask questions, seek guidance and receive feedback:

‘Our supervisor was the one who arrived only on Monday. So, the majority of the days we went without supervision, without guidance.’ (Participant 5, male, student SLT)

‘We only get to experience the hospital site for a short time … the supervisors expect us to already know everything.’ (Participant 2, female, student SLT)

#### Supervisors workload constraints

Six students commented on supervisors’ heavy workloads, which reduced their availability to engage with students. They recognised that clinical supervisors had competing responsibilities that limited the time and attention that could be devoted to student learning:

‘The staff … employed permanently … they had to do their own work … sometimes you go to a person to ask for something but they can’t help you because they’re busy with their own things.’ (Participant 11, female, student SLT)

#### Resource limitations

Nine students described how limited assessment tools and therapeutic materials made it difficult to carry out evidence-based practice, often forcing them to rely on improvised or online resources:

‘We had to use YouTube to look up information … we didn’t have the tools or enough information for managing the cases properly.’ (Participant 1, male, student SLT)

These resource and supervision constraints were especially pronounced in under-resourced settings, contributing to anxiety and limiting opportunities for hands-on learning.

### Theme 3: Student experiences of clinical supervision

Students’ reflections highlighted how clinical supervision shaped their emotional well-being, confidence and professional identity. Their experiences showed that supervision is not only instructional but deeply relational.

#### Growth through supportive relationships

Six students attributed much of their growth to supervisors who were approachable, encouraging and willing to guide them through clinical challenges. Interaction with supervisors and peers from other universities also broadened their clinical perspectives. Clinical sites host students from different universities:

‘They helped me grow as a student and as a clinician … I interacted with different speech therapists from different universities with different knowledge.’ (Participant 11, female, student SLT)

#### Emotional reassurance

Students (*n* = 8) deeply valued supervisors who fostered a safe, non-judgemental learning environment:

‘She was always there to provide support. Even when I didn’t know what to do, she’d guide me without making me feel incompetent.’ (Participant 8, male student SLT)

#### Avoidance of help-seeking in unsupportive environments

In contrast, unsupportive supervisory behaviours led some students (*n* = 4) to withdraw and avoid asking for help, which undermined their confidence:

‘We end up not asking some questions because we were rejected by the supervisor … we were always shouted at … it reduces our confidence and we were afraid to ask things.’ (Participant 1, male, student SLT)

#### Developing adaptability across settings

Exposure to multiple service settings, such as hospitals, special schools, autism centres and mainstream schools, helped students (*n* = 6) adapt to different clinical expectations and patient needs:

‘I was placed at different schools and hospitals … each place has its own way of doing things, so you learn to do things differently.’ (Participant 8, male student SLT)

#### Negative emotional experiences

Students (*n* = 9) also described anxiety and stress resulting from inconsistent or punitive supervision:

‘I felt like the supervisors expected more of us than what we were prepared for. That added pressure made me more anxious.’ (Participant 2, female, student SLT)

These experiences highlight that clinical supervision influences not only students’ skill development but also their emotional resilience, confidence, and willingness to engage.

## Discussion

This study identified three interconnected themes shaping speech-language therapy students’ experiences of clinical supervision: (1) facilitators of clinical supervision, (2) barriers to clinical supervision, and (3) student experiences of clinical supervision. Interpreting these findings through Proctor’s (1986) model highlights how formative, normative and restorative supervisory functions intersect within the South African clinical training context. The theme *facilitators of clinical supervision* demonstrated how structured guidance, actionable feedback, access to clinical resources and preparatory tutorials contributed to students’ clinical development. Constructive feedback strengthened the formative function by helping students recognise errors, refine assessment procedures and improve therapeutic decision-making. Tutorials further supported formative learning by offering structured opportunities to consolidate theory and practise clinical essential skills. These findings align with literature noting that specific, high-quality feedback and supported learning structures reinforce skill acquisition and professional growth (Burgess et al., 2014; Rothwell et al., 2021; Weallans et al., 2022). Access to assessment tools, protocols and clinical articles also reinforced the normative function by clarifying expectations, promoting consistent standards and guiding students in delivering assessments and interventions appropriately. The restorative function of supervision was highlighted through students’ experiences of emotional reassurance, patience and a sense of psychological safety. Supportive supervisors enabled students to seek help without fear of judgement, strengthening confidence and encouraging active clinical participation. This is consistent with existing literature emphasising the role of emotional safety in facilitating engagement and resilience during clinical learning (Rothwell et al., 2021; Thyness et al., 2022). In contrast, the theme *barriers to clinical supervision* revealed how inconsistent supervision, high supervisor workload and resource limitations weakened all three supervisory functions. When supervisors were present only intermittently or were unable to engage because of competing clinical demands, opportunities for feedback, modelling, and clarification diminished. This constrained the formative function by limiting practice-based learning. High supervisor workloads and the absence of necessary clinical materials disrupted the normative function, as students lacked access to guidance on expected standards or appropriate procedures. These findings align with prior research showing that time pressure, understaffing, and resource shortages reduce the capacity for effective supervision in busy healthcare environments (Jelinek et al., 2010; Kenny & Allenby, 2013). Students also described increased anxiety and uncertainty in such contexts, indicating weakened restorative support, as supervision did not consistently provide emotional containment or reassurance. The third theme, *student experiences of clinical supervision*, illustrated how supervision influenced professional identity formation, confidence and adaptability. Positive experiences were characterised by supportive supervisory relationships, exposure to varied clinical contexts, and opportunities to develop independence. These experiences align with literature highlighting the value of diverse clinical exposure in fostering adaptability and cultural responsiveness (Parkin et al., 2025). In contrast, students who felt judged or unsupported described withdrawing from help-seeking, increased anxiety, and relying more heavily on peers for guidance. These responses highlight the relational component of supervision and demonstrate how a lack of restorative support can undermine learning, consistent with concerns raised by Thyness et al. (2022) regarding psychological safety. These contextual barriers reflect challenges commonly reported in the Eastern Cape, where infrastructure limitations, staffing shortages, and high service demands shape supervision practices. Although global supervision frameworks remain useful, the findings suggest that supervision in such environments must be adaptable and responsive to real-world constraints while still upholding the formative, normative, and restorative functions outlined in Proctor’s model. Notably, the data also show that high-quality supervision is achievable even in low-resource settings when supervisors provide structured tutorials, timely feedback and a supportive presence. These foundational elements, though basic, had a significant positive impact on students’ confidence, competence and perceived learning, demonstrating that effective supervision need not be resource-intensive when implemented through a student-centred and relationally attuned approach. Overall, the findings emphasise that effective clinical supervision requires balance across the formative, normative and restorative functions. Supportive, consistent supervision enhanced students’ confidence, competence and emotional well-being, whereas inconsistent or punitive supervision hindered skill development and reduced engagement. Strengthening supervisory structures, ensuring resource availability and promoting emotionally safe supervisory relationships are therefore essential for improving the clinical education of speech-language therapy students in under-resourced South African contexts.

## Strengths and limitations

A key strength of this study lies in its use of qualitative methods to capture students’ lived experiences of clinical supervision, perspectives often missing in the literature on health professions education. The use of Proctor’s model (Proctor, 1986) provided a structured yet flexible framework for interpreting findings within a real-world clinical training context. The study’s focus on the Eastern Cape adds important depth to our understanding of supervision in under-resourced regions. However, the study is limited by its focus on one province, one university and a relatively small sample size, which may constrain transferability to other contexts. Additionally, the voices of clinical supervisors and institutional policymakers were not included. Including these perspectives could have offered a more comprehensive understanding of the structural and operational challenges within the supervision framework.

## Implications and recommendations

This study highlights the need for a more consistent and holistic approach to clinical supervision that integrates all three of Proctor’s domains. Training institutions are encouraged to prioritise supervisor development programmes to ensure that supervision supports not only skill acquisition (formative) but also ethical growth (normative) and emotional well-being (restorative). Although the HPCSA outlines minimum training requirements for supervision, student feedback in this study indicates variability in how these expectations are implemented across training sites. Strengthening institutional oversight and fostering partnerships with clinical sites may, therefore, help promote more consistent supervision practices and improve equity in the quality of students’ learning experiences. Improvements in infrastructure, such as access to updated assessment tools, reliable internet connectivity and appropriate clinical materials, are also necessary to create more enabling learning environments, particularly in under-resourced contexts. Future research should include the perspectives of supervisors, university staff and training site managers to provide a fuller picture of the supervisory ecosystem. Multi-site or cross-provincial studies would help build a broader understanding of clinical training realities and highlight shared challenges or context-specific dynamics.

## Conclusion

This study explored the perspectives of speech-language therapy students on the clinical support they received during training in South Africa. Using Proctor’s model (Proctor, 1986) as a framework, the findings highlight the importance of balancing educational, ethical and emotional dimensions of supervision to foster professional development. While students appreciated structured learning opportunities and supportive supervisors, their experiences were often uneven across placement sites. Inadequate resources, inconsistent supervision and contextual barriers in under-resourced settings hindered optimal clinical learning. These findings point to the need for a more standardised, equitable, and context-sensitive approach to clinical supervision that recognises and responds to the real-world constraints of training in the South African higher education system.

## References

[CIT0001] Abrahams, F., & Khoza-Shangase, K. (2025). South African Speech-Language Therapy and Audiology students’ experiences of academic and clinical curriculum transformation. *South African Journal of Communication Disorders*, 72(1), 1086. 10.4102/sajcd.v72i1.1086 PMC12135758 40459123

[CIT0002] Attrill, S., Lincoln, M., & McAllister, S. (2015). International students in speech-language pathology clinical education placements: Perceptions of experience and competency development. *International Journal of Speech-Language Pathology*, 17(3), 314–324. 10.3109/17549507.2015.1016109 25764340

[CIT0003] Babbie, E.R. (2020). *The practice of social research* (15th ed.). Cengage.

[CIT0004] Bailey, R. (2021). Speech-language pathologist. In L. McIlwraith & J. McCormack (Eds.), *Collaboration* (pp. 105–122). Routledge. 10.4324/9781003233688-12

[CIT0005] Balay-odao, E.M., Almazan, J., Mesde, J., Bajet, J.B., Alquwez, N., Danglipen, C.C., & Cruz, J.P. (2025). Influence of clinical supervision and ethical sensitivity on the compassion competence of nursing students. *Health Professions Education*, 11(3), 11. 10.55890/2452-3011.1352

[CIT0006] Biggs, J., & Tang, C. (2014). Constructive alignment: An outcomes-based approach to teaching anatomy. In L. Chan & W. Pawlina (Eds.), *Teaching anatomy: A practical guide*. Springer. 10.1007/978-3-319-08930-0_4

[CIT0007] Braun, V., & Clarke, V. (2006). Using thematic analysis in psychology. *Qualitative Research in Psychology*, 3(2), 77–101. 10.1191/1478088706qp063oa

[CIT0008] Burgess, A., Oates, K., Goulston, K., & Mellis, C. (2014). First year clinical tutorials: Students’ learning experience. *Advances in Medical Education and Practice*, 5, 451–456. 10.2147/AMEP.S73395 25489253 PMC4257052

[CIT0009] Byrne, E., Brugha, R., Clarke, E., Lavelle, A., & McGarvey, A. (2015). Peer interviewing in medical education research: Experiences and perceptions of student interviewers and interviewees. *BMC Research Notes*, 8(1), 513. 10.1186/s13104-015-1484-2 26423420 PMC4588247

[CIT0010] Creswell, J.W., & Creswell, J.D. (2017). *Research design: Qualitative, quantitative, and mixed methods approaches*. Sage.

[CIT0011] Creswell, J.W., & Poth, C.N. (2016). *Qualitative inquiry and research design: Choosing among five approaches*. Sage.

[CIT0012] Falender, C.A., & Shafranske, E.P. (2014). Clinical supervision: The state of the art. *Journal of Clinical Psychology*, 70(11), 1030–1041. 10.1002/jclp.22124 25220545

[CIT0013] Farooq, M.B., & De Villiers, C. (2017). Telephonic qualitative research interviews: When to consider them and how to do them. *Meditari Accountancy Research*, 25(2), 291–316. 10.1108/MEDAR-10-2016-0083

[CIT0014] Guest, G., Namey, E., & Chen, M. (2020). A simple method to assess and report thematic saturation in qualitative research. *PLoS One*, 15(5), e0232076. 10.1371/journal.pone.0232076 32369511 PMC7200005

[CIT0015] Hardy, J., Lewis, A., Walters, J., Hill, A.E., Arnott, S., Penman, A., Attrill, S., Nicholls, R., & Hewat, S. (2021). Reflections on clinical education by students and new graduates: What can we learn? *Journal of Clinical Practice in Speech-Language Pathology*, 23(3), 146–150. 10.1080/22087168.2021.12370334

[CIT0016] Hendricks, S., Cartwright, D.J., & Cowden, R.G. (2021). Clinical supervision in South Africa: Perceptions of supervision training, practices, and professional competencies. *South African Journal of Science*, 117(3–4). Retrieved from https://www.ajol.info/index.php/sajsci/article/view/219822

[CIT0017] Jelinek, G.A., Weiland, T.J., & Mackinlay, C. (2010). Supervision and feedback for junior medical staff in Australian emergency departments: Findings from the emergency medicine capacity assessment study. *BMC Medical Education*, 10(1), 74. 10.1186/1472-6920-10-74 21044342 PMC2987934

[CIT0018] Kenny, A., & Allenby, A. (2013). Implementing clinical supervision for Australian rural nurses. *Nurse Education in Practice*, 13(3), 165–169. 10.1016/j.nepr.2012.08.009 22980923

[CIT0019] Khoza-Shangase, K., & Mophosho, M. (2021). Language and culture in speech-language and hearing professions in South Africa: Re-imagining practice. *South African Journal of Communication Disorders*, 68(1), 1–9. 10.4102/sajcd.v68i1.793 PMC8252163 34082547

[CIT0020] King, C., Edlington, T., & Williams, B. (2020). The ‘ideal’ clinical supervision environment in nursing and allied health. *Journal of Multidisciplinary Healthcare*, 13, 187–196. 10.2147/JMDH.S239559 32110033 PMC7034973

[CIT0021] Kolb, D.A. (1984). *Experiential learning: Experience as the source of learning and development*. Prentice-Hall.

[CIT0022] Litherland, G., Schulthes, G., Cowles, C., & Ewe, E. (2023). The proctor model of clinical supervision: An introduction for professional counselors. *Journal of Counselor Preparation and Supervision*, 17(5), 4. 10.7290/tsc05i2is

[CIT0023] McKim, C. (2023). Meaningful member-checking: A structured approach to member-checking. *American Journal of Qualitative Research*, 7(2), 41–52.

[CIT0024] Müller, J., Reardon, C., Coetzee, F., Bester, J., Dube, K., Hanekom, S., Du Plessis, E., & Couper, I. (2022). Transformative learning through participation: Experiences at a rural clinical training site in South Africa. *BMC Medical Education*, 22(1), 183. 10.1186/s12909-022-03233-w 35296325 PMC8928645

[CIT0025] Mupawose, A., Adams, S., & Moonsamy Maistry, S. (2021). Facilitators of and barriers to clinical supervision of speech-language pathology students in South Africa: A pilot study. *African Journal of Health Professions Education*, 13(1), 23–28. 10.7196/AJHPE.2021.v13i1.1216

[CIT0026] Nagdee, N., Sebothoma, B., Madahana, M., Khoza-Shangase, K., & Moroe, N. (2022). Simulations as a mode of clinical training in healthcare professions: A scoping review to guide planning in speech-language pathology and audiology during the COVID-19 pandemic and beyond. *South African Journal of Communication Disorders*, 69(2), a905. 10.4102/sajcd.v69i2.905 PMC9452917 36073069

[CIT0027] Nowell, L.S., Norris, J.M., White, D.E., & Moules, N.J. (2017). Thematic analysis: Striving to meet the trustworthiness criteria. *International Journal of Qualitative Methods*, 16(1), 1609406917733847. 10.1177/1609406917733847

[CIT0028] O‘Brien, A.T., McNeil, K., & Dawson, A. (2019). The student experience of clinical supervision across health disciplines – Perspectives and remedies to enhance clinical placement. *Nurse Education in Practice*, 34, 48–55. 10.1016/j.nepr.2018.11.006 30458410

[CIT0029] Panda, S., Dash, M., John, J., Rath, K., Debata, A., Swain, D., Mohanty, K., & Eustace-Cook, J. (2021). Challenges faced by student nurses and midwives in clinical learning environment – A systematic review and meta-synthesis. *Nurse Education Today*, 101, 104875. 10.1016/j.nedt.2021.104875 33774528

[CIT0030] Parkin, N., Pearce, K., Stengewis, R., & Drummond, C. (2025). Learning in a contextually complex rural clinical placement. *Advances in Health Sciences Education*, 31(1), 239–258. 10.1007/s10459-025-10443-6 40471502 PMC12929316

[CIT0031] Pillay, M., & Kathard, H. (2018). Audiology and speech-language pathology: Practitioners’ reflections on indigeneity, disability and neo-colonial marketing. *Disability and the Global South*, 5(2), 1365–1384.

[CIT0032] Pollens, R. (2020). Facilitating client ability to communicate in palliative end-of-life care: Impact of speech–language pathologists. *Topics in Language Disorders*, 40(3), 264–277. 10.1097/TLD.0000000000000220

[CIT0033] Pope, K., Barclay, L., Dixon, K., & Kent, F. (2023). Models of pre-registration student supervision in allied health: A scoping review. *Focus on Health Professional Education: A Multi-Professional Journal*, 24(2), 27–62. 10.11157/fohpe.v24i2.559

[CIT0034] Proctor, B. (1986). *Supervision: A co-operative exercise in accountability, enabling and ensuring* (pp. 21–24). Leicester National Youth Bureau and Council for Education and Training in Youth Work.

[CIT0035] Quigley, D., Loftus, L., McGuire, A., & O‘Grady, K. (2020). An optimal environment for placement learning: Listening to the voices of speech and language therapy students. *International Journal of Language & Communication Disorders*, 55(4), 506–519. 10.1111/1460-6984.12533 32189425

[CIT0036] Richards, K.A.R., & Hemphill, M.A. (2018). A practical guide to collaborative qualitative data analysis. *Journal of Teaching in Physical Education*, 37(2), 225–231. 10.1123/jtpe.2017-0084

[CIT0037] Rocha, J., Santos, C., Peixoto, V., Maia, F., & Alegria, R. (2020). Speech language pathology Clinical Education: Perceptions and experiences of clinical educators and students. *Revista de Investigación en Logopedia*, 10(2), 123–133. 10.5209/rlog.65677

[CIT0038] Rothwell, C., Kehoe, A., Farook, S.F., & Illing, J. (2021). Enablers and barriers to effective clinical supervision in the workplace: A rapid evidence review. *BMJ Open*, 11(9), e052929. 10.1136/bmjopen-2021-052929 PMC8479981 34588261

[CIT0039] Saxon, R.L., Gray, M.A., & Oprescu, F.I. (2014). Extended roles for allied health professionals: An updated systematic review of the evidence. *Journal of Multidisciplinary Healthcare*, 7, 479–488. 10.2147/JMDH.S66746rocha 25342909 PMC4206389

[CIT0040] Sheepway, L., Lincoln, M., & McAllister, S. (2014). Impact of placement type on the development of clinical competency in speech-language pathology students. *International Journal of Language & Communication Disorders*, 49(2), 189–203. 10.1111/1460-6984.12059 24182204

[CIT0041] Sims, D., Zingela, Z., Mokhachane, M., Botha, G., Mawela, D., Singaram, V., Baatjes, K., Green-Thompson, L., & Begg, K. (2025). Medical education, reflections and perspectives from South Africa: A review. *BMC Medical Education*, 25(1), 370. 10.1186/s12909-025-06910-8 40075353 PMC11900656

[CIT0042] Snowdon, D.A., Sargent, M., Williams, C.M., Maloney, S., Caspers, K., & Taylor, N.F. (2019). Effective clinical supervision of allied health professionals: A mixed methods study. *BMC Health Services Research*, 20(1), 2. 10.1186/s12913-019-4873-8 31888611 PMC6937808

[CIT0043] Terry, D., Nguyen, H., Perkins, A., & Peck, B. (2020). Supervision in healthcare: A critical review of the role, function and capacity for training. *Universal Journal of Public Health*, 8(1), 1–14. 10.13189/ujph.2020.080101

[CIT0044] Thyness, C., Steinsbekk, A., & Grimstad, H. (2022). Learning from clinical supervision – A qualitative study of undergraduate medical students’ experiences. *Medical Education Online*, 27(1), 2048514. 10.1080/10872981.2022.2048514 35249473 PMC8903767

[CIT0045] Weallans, J., Roberts, C., Hamilton, S., & Parker, S. (2022). Guidance for providing effective feedback in clinical supervision in postgraduate medical education: A systematic review. *Postgraduate Medical Journal*, 98(1156), 138–149. 10.1136/postgradmedj-2020-139566 33563716

[CIT0046] Weaver, K. (2007). Ethical sensitivity: State of knowledge and needs for further research. *Nursing Ethics*, 14(2), 141–155. 10.1177/0969733007073694 17425144

[CIT0047] Willie, M.M., & Maqbool, M. (2023). Access to public health services in South Africa’s rural Eastern Cape Province. *Applied Sciences Research Periodicals*, 1(2), 35–54. 10.2139/ssrn.4405870

[CIT0048] Wilson, H.M., Davies, J.S., & Weatherhead, S. (2016). Trainee therapists’ experiences of supervision during training: A meta-synthesis. *Clinical Psychology & Psychotherapy*, 23(4), 340–351. 10.1002/cpp.1957 25917244

[CIT0049] Yang, K., Nisbet, G., & McAllister, L. (2017). Students’ experiences and perceptions of interprofessional supervision on placement. *International Journal of Practice-based Learning in Health & Social Care*, 5(2), 1–18. 10.18552/ijpblhsc.v5i2.361

